# Water Supply and Sewerage Problem of Buffalo1President’s address at the seventy-ninth Annual Meeting of the Medical Society of the County of Erie, January 9, 1900.

**Published:** 1900-03

**Authors:** J. B. Coakley

**Affiliations:** Buffalo, N. Y.


### WATER SUPPLY AND SEWERAGE PROBLEM OF BUFFALO.[1]

By J. B. COAKLEY, M. D., Buffalo, N. Y.

IN OCTOBER, 1893, the Board of Councilmen appointed a committee consisting of Councilmen Hanrahan, Sandrock and myself to investigate the desirability of constructing a sewer in Tifft Street, and to ascertain whether Buffalo River, Niagara River and other waterways were being contaminated with sewage. The committee conferred with at least half a dozen city officials, who were in office at that time, and a verbal report was made to the board, which protested against sewering into sluggish waterways within the city limits, which have outlets into Niagara River, and especially against sewering into Buffalo River. But we failed in our efforts to make the city officials realise that there was any real danger to the water supply, for they insisted that the entire contents of Buffalo River were drained into Black Rock harbor and did not reach Niagara River. Nevertheless in February and March of the following year (1894) there was an outbreak of typhoid fever, and the officials were compelled to admit that the contamination of the water supply was due to water drawn from the Niagara River near the American shore, known as the Bird Island intake.

1. President's address at the seventy-ninth Annual Meeting of the Medical Society of the County of Erie, January 9, 1900.

In April, 1895, a committee of the Board of Aldermen reported on the water supply of the city. The report consists of a statement of the conditions existing at the time, and of certain recommendations. The latter were, briefly, as follows:

First—To enlarge the tunnel.

Second—To construct a second tunnel parallel to the existing tunnel.

Third.—To extend the old tunnel from the Bird Island Pier out into the river to clear water, by means of a pipe laid in an excavation cut in the rock.

Fourth—To obtain a new 30,000,000 gallon engine to be placed at the lower pumping station, and also four new boilers to pump direct into the new reservoir, and to have the three existing Worthington engines rebuilt.

The committee reported that "prompt action was necessary to protect the City from a great catastrophe—either in the shape of a famine or an accident to the plant which is absolutely unprotected." An explosion at the pumping station would probably destroy the entire apparatus and the city would be without water. A fire at the pumping station would bring about the same dire result. "The danger," said the committee, "is too great to be ignored, the responsibility too grave to be lightly passed over." In December, 1895, Asst.-Supt. Knapp made a report to the board of Public Works upon pumping plans, and upon the location of the new tunnel and intake. And in October, 1896, Mr. Rudolph Hering, of New York, an expert engineer, made a lengthy report, in which he put forward very much the same recommendations as Asst.-Supt. Knapp. These reports were published in the newspapers at the time and are too long for reproduction here.

In the spring of last year I happened to look out of a window on an upper floor of the Prudential Building. The rainfall having been heavy, Buffalo River was very full, and there being strong southeasterly winds, it was swept out into Niagara River to a very considerable extent. The line of demarkation was easily apparent in consequence of the difference in color between the water of Buffalo River and that of the lake. Being strongly impressed with the belief that the contents of the former reached the inlet pier—the place at which our drinking water is obtained—I went to the office of the Mayor to request him to look at the spot where Buffalo River joins Niagara River from some high building before the effects of the heavy rainfall and the wind had disappeared. His Honor was away from the city, but I mentioned the facts to his secretary, Mr. Fleming, and also to our health officer, Dr. Wende, both of whom afterward told me that they had observed the same condition of the water. On March 30, 1896, Engineer Knapp noticed that the roily water of Buffalo River reached a point 20 or 30 feet west of the inlet pier in the Niagara River, and it continued to do so for four hours. Engineer Knapp put this incident on his record at the time. The probability is that proof of Buffalo River water entering Niagara River can be obtained at any time after heavy rains. The time for action has arrived. Everything, including the land and the legislation necessary to acquire title to the same, is now in such condition that the Council can order the tunnel, the extension and the excavation commenced without unnecessary delay. The total cost of the work has been estimated at about $500,000, and while this may seem a large sum, we must remember that good water will contribute more to the health and happiness of the human race than any other single article that can be mentioned. Water is the best and cheapest of all medicines and in spending money to obtain a satisfactory supply we shall, I feel sure, contribute to the general prosperity of this city. All other improvements can better afford to wait than this one, for, apart from hygienic reasons any defect in the quantity or the quality of the water during the Pan-American Exposition would give Buffalo an unenevi-able notoriety for many years to come.

Buffalo appears to require (including a large quantity wasted) about 100,000,000 gallons of water every twenty-four hours, and I know of no process by which the consumption can be reduced, without injury to the community. As it is, few people drink enough water, and nobody, outside our own profession, does too much bathing. Now, a few words about filtration. I ask you to bear in mind that the filtration of water for Buffalo is a very different matter from the filtration of water for European cities. While few of the latter use more than twenty-five gallons per day per head of population, New York uses sixty and Philadelphia ninety gallons per day. The cost of filtering the whole of the water for our city, including the interest upon the investment, has been estimated by Mr. Rudolph Hering at not less than $6 per 1,000,000 gallons. It would seem as though filtered water is misused when applied to such purposes as to lay dust, to generate steam and to flush sewers, for all these processes lead to repollution, without any benefit whatever. A duplicate supply, one for domestic and the other for public purposes, would be a great advantage; but I am afraid that such a plan is outside the range of practical politics at present. As to filters: The American or mechanical system of filtration is more rapid than the European method. The former consists of the formation of an inorganic slime upon the small grains of sand. This is created by adding to the water, as it goes into the filter, something less than one grain of alum to each gallon of water. The lime in the water decomposes this alum, forming an aluminum hydrate, which settles upon the sand at the bottom of the filtering bed. The cost of this form of filter is about $70,000 per acre, which ought to produce more than 2,000,000 gallons of wholesome water every twenty-four hours. A filtering plant requires constant attention and cleaning, the cleansing process consisting of "reversing the flow of water until the flow is clear."

Permit me to point out that one of the most satisfactory results of patient scientific investigation is that public opinion is aroused, and puts itself in evidence by the enactment of city ordinances and the passage of statutes by the state. How long it will be before we impress the laity with the absolute necessity of improving the quality of the water I cannot say, but we have now succeeded, I believe, in making it generally understood that typhoid fever is, in the great majority of cases, due to contaminated drinking water. The bacillus typhosus is probably the greatest disease-producing microörganism in human excrement, the extent of its vitality is known with moderate accuracy, and extremes of cold do not deprive it of its pathogenic power. If favored by a current in the lake of from two to four miles an hour, it might travel from one end of Lake Erie to the other, eventually reaching Buffalo in full possession of all its death-dealing qualities.

The idea that water is purified by the oxidation of organic matter as a result of the direct action of atmospheric oxygen, is a fallacy of a wide-spread character. The experiments of Profs. Leeds and Mason, and the practical experience of Newburyport, Mass., in 1893, have shown the error of this doctrine so conclusively that, in a paper upon The relation of impure water to disease—published in 1897, Dr. Carpenter, assistant bacteriologist of this city, said: "The adage, 'No river is long enough to purify itself' would seem to have been about proved."

A very small amount of consideration would lead one to the conclusion that concerted action upon the part of Buffalo, Erie, Cleveland and Detroit would result in some measures being adopted which would materially reduce the amount of refuse, garbage, etc., thrown into the lake; but this suggestion was made half a dozen years ago, and has been repeated many times without anything being done, apparently because no body is willing to take the initiative. That being so, the question before us is, What means can be employed by the City of Buffalo, without extraneous aid, to improve the condition of its water? The Health Department is doing all that can be done in the way of chemical and bacteriological examinations. I am under an obligation to this department and also to the Department of Public Works for a considerable part of the information I am able to place before you, and I wish to express my sincere thanks to the gentlemen in both departments who have so kindly given me their valuable assistance. Without their aid, this paper would have contained only a modicum of facts.

Drinking water, to be fit for human consumption, ought to be free from pathogenic microörganisms, and it ought also to be in accordance with a well-known chemical standard; it need not, of course, be absolutely pure, but, on the contrary, it should contain a small quantity of mineral salts in solution, as well as gases in solution, and water-purifying organisms. It is obvious, I need hardly say, that water may be in good condition, as far as chemical analysis will show, may yet be unfit for drinking purposes by reason of the presence of pathogenic organisms. It is generally admitted, I assume, that whenever the opinions of the chemist and the bacteriologist differ as to the quality of the water, the public should boil it before drinking it. A statement of the technical methods by which water is shown to be potable or unfit for drinking purposes would be out of place here, even if the time were at my disposal. I propose to say a few words as to the condition of the water at various points; but before doing so, I beg leave to place before you the desiderata of a satisfactory water supply, as laid down by Dr. William C. Krauss, of this city, at a meeting of the American Microscopical Society in 1896.

"First—The water supply should not in any possible way be liable to pollution or contamination from the sewage of any other community.

Second—The sewage should not be emptied into any water course not having a current of three to five miles per hour, and then the sewage entrance should be at a distance one mile or more from the intake.

"Third—When the water supply of any city is a navigable stream the water should be sand filtered before being pumped into the city reservoirs or water mains.

"Fourth—For ordinary drinking purposes the water should not be taken in its primitive or raw state, but should be either filtered, boiled or distilled and aerated.

"Fifth— That not only chemical, but bacteriological examinations of the water should be made, at least once weekly, to determine its character as a safe or dangerous water for domestic use, and where contamination is shown to exist, the services of an engineer should be enlisted to detect, if possible, the cause and origin of such contamination."

At the request of Dr. Wende, Dr. Carpenter went with me on December 11, 1899, to obtain samples of water at various places. These were taken both at the surface and also six feet below the surface. They have been examined by the City Chemist and by the Bacteriologist. The water obtained from Buffalo River, opposite the life-saving station is described as "not fit for drinking purposes"; that obtained at the junction of the Buffalo and Niagara Rivers, opposite the lighthouse, is reported to be "bad water;" the water taken from the Niagara River, opposite the Bird Island Inlet, contained 3,773 bacteria per cubic centimeter, and is, therefore, bad water as compared with that taken from the tap on December 11th, which contained only 386 bacteria per cubic centimeter. The sample obtained at the Bird Island Inlet (Niagara River shore line) is described as "bad water" by the bacteriologist. Upon the other hand the water taken from the lake near the Horseshoe Reef known as Emerald Channel, was "good water," and contained only 373 bacteria per cubic centimeter. It is described by the chemist as "slightly turbid," although "good drinking water." I am well aware, of course, that to every pathogenic microörganism in water, there must be almost 100 which are nonpathogenic; but nothing would be gained by further reference to the general bacteriology of water at this time.

As Lake Erie is shallow, the average depth not being over 100 feet, the water from November to April or May, often contains a good deal of sand, which, when taken into the stomach, occasionally produces mild diarrhea, sometimes alarming to the sufferers and the newspapers, but otherwise unimportant.

Lake Erie now receives the refuse of so many cities that fully 1,000,000 people contribute to its contamination; and as the water for our city comes from the Niagara River—the so-called "focus point" of the great lakes—Buffalo is likely to suffer to an alarming extent, as the cities continue to grow and the refuse deposited in the lake increases in quantity. In my opinion it is the bounden duty of every city to do something toward the purification of its sewage, either by sterilisation or by some other process.

I want to say that our method of polluting streams and lakes by sewage is a growing evil and a very serious one. In many European countries it is forbidden by law and in this country such laws are imperatively required in order to preserve sufficient unpolluted water for the large cities. I shall return to this point later on. I wish now to draw your attention to the fact that our water is in danger of being additionally contaminated by the workers at the new steel plant, which is to be erected at Stony Point. We are told that a very large number of persons are to be employed there, and it is, in my opinion, the duty of the city to obtain some guarantee that this addition to the population will not involve increased contamination of the water. The condition is quite bad enough without any increase in the sewage. The Health Department has quite frequently told us that "the water is in poor condition," or that "there is evidence of contamination." Moreover, the statistics of typhoid demonstrate conclusively that while Buffalo compares favorably with other cities upon this continent, the number of cases of typhoid fever and the typhoid death-rate are far too high. I mean that, with our present knowledge the mortality rate from this disease can be reduced to 20 per 100,000 of population, provided we can induce the public to insist upon the necessary money being spent upon certain sanitary measures. Before mentioning these, I ask you to look at the number of cases of typhoid during the last six years. The dath-rate per 100,000 of population has varied, since 1894, between 19½ and 60.

Number of cases of typhoid fever and of deaths from that disease since 1894:

| | Cases. | Deaths. |
|---|---|---|
| 1894 | 1,088 | 185 |
| 1895 | 397 | 98 |
| 1896 | 274 | 68 |
| 1897 | 201 | 63 |
| 1898 | 280 | 98 |
| 1899 | 211 | 87 |

Upon November 24th of last year (1899) Dr. Gram, of the Health Department, wrote to me that in 1898, 110 cases of typhoid were of soldiers sent home from the war, with 11 deaths.

The stirring up of Lake Erie by storms may have certain drawbacks, but it also has decided advantages. Dr. Drown, until recently a resident of Boston, has shown that the amount of oxygen in solution in the water of natural ponds and lakes decreases toward the bottom, until, when the bottom is finally reached, it is, in many cases, almost nil. In such cases, the aerobic microörganisms must inevitably succumb to the anaerobic, which latter, including the bacillus typhosus, have a free field in which to develop to the utmost limit of luxuriance. The stirring-up which the storms produce will bring the beneficent action of oxygen and aerobic life to bear upon injurious microörganisms, which, otherwise would never experience such influence. Upon the other hand, while a gale continues, the deposited sewage of a considerable period of calm is thoroughly mixed with the whole body of water. If, however, you consider the facts in their entirety, you will, I am very confident, agree with me in the following assertion: no pollution of a lake or river by unpurified sewage is justifiable and a period has now been reached when the feasibility of treating the sewage of Buffalo by some scientific process ought to be considered. But I am specially anxious to impress upon you that, granting the pollution of the lake by other cities, we would nevertheless obtain much better water by constructing a new tunnel out into the lake than by taking it from the Niagara River, as we do at present.

I now request your attention to the question of sewage disposal. Several years ago Dr. Krauss pointed out that an act of Congress was urgently required prohibiting the "dumping" of untreated sewage in the lake or in any waterway which flows into the lake. But nothing has been done, and Cleveland, Buffalo and other cities all send their sewage without any attempt at purification, to mingle with the water of Lake Erie. These same cities take their drinking water from the same lake. In other words, we, citizens of Buffalo, numbering over 350,000, complacently drink raw water which must at times, contain raw sewage! In reality, the surprising part of this state of affairs is that typhoid fever has not caused more than 185 deaths in any one year since 1893.

The sewage system of Buffalo, however, is about to be improved, although the sewage will still continue to flow into the lake without any purification. For the following information I am indebted to the City Engineer, Mr. Bardol, and his assistant, Mr. Buttolph.

Under plans and specifications of the Engineer's office, contracts are about to be made for the drainage of the Hamburg Canal and its tributaries which, when completed, will about tax the present trunk sewer—the so-called Swan Street sewer—to its full capacity. In the districts south of the Hamburg Canal, and on both sides of Buffalo River, another system is necessary which will involve the construction of another trunk sewer, as well as several sewer-pumping plants. The number of sewers entering Buffalo River is seventeen; the number draining into the Hamburg Canal is fourteen; eleven flow into the Ohio Basin Slip, and nine into Cazenovia Creek. These drain a district having a population of 12,000 south of Buffalo River, and 26,000 north of that river—total 38,000. Buffalo River is further contaminated by receiving the drainage from foundaries (three), two chemical works and two oil refineries, one coach-glue factory, one linseed oil factory and the refuse from 24 elevators and 12 freight-houses, which employ 3,000 men.

I wish to urge most strongly that before any new plans are laid out for building sewers the whole question of sewage disposal ought to be thoroughly investigated by the city authorities.

In conclusion, then, I ask for the following measures as imperatively demanded with all reasonable speed.

First.—The construction of the new tunnel to the lake and another pumping-station at the foot of Porter Avenue, and the purchase of new engines sufficient for the needs of the city, as advised by the aldermanic committee and the engineers.

The position of Buffalo in reference to its water plant is anomalous, for, while we have only one pumping plant, Boston has four pumping plants, New York has three, Chicago has eight and Philadelphia has ten.

Second—The immediate consideration of all questions connected with the disposal of the sewage, with a view to the abatement of the existing evil.

Third—The positive insistence by the city of some system of drainage at the new steel works that will effectually prevent any additional comtamination of the drinking water.

339 DELAWARE AVENUE.

